# Parallel imaging with phonon microscopy using a multi-core fibre bundle detection

**DOI:** 10.1016/j.pacs.2023.100493

**Published:** 2023-04-24

**Authors:** Rafael Fuentes-Domínguez, Mengting Yao, William Hardiman, Salvatore La Cavera III, Kerry Setchfield, Fernando Pérez-Cota, Richard J. Smith, Matt Clark

**Affiliations:** Optics and Photonics Group, University of Nottingham, University Park, Nottingham, NG7 2RD, UK

**Keywords:** Picosecond ultrasonics, Phonon microscopy, Time-domain Brillouin scattering, Parallel measurements

## Abstract

In this paper, we show a proof-of-concept method to parallelise phonon microscopy measurements for cell elasticity imaging by demonstrating a 3-fold increase in acquisition speed which is limited by current acquisition hardware. Phonon microscopy is based on time-resolved Brillouin scattering, which uses a pump–probe method with asynchronous optical sampling (ASOPS) to generate and detect coherent phonons. This enables access to the cell elasticity via the Brillouin frequency with sub-optical axial resolution. Although systems based on ASOPS are typically faster compared to the ones built with a mechanical delay line, they are still very slow to study real time changes at the cellular level. Additionally, the biocompatibility is reduced due to long light exposure and scanning time. Using a multi-core fibre bundle rather than a single channel for detection, we acquire 6 channels simultaneously allowing us to speed-up measurements, and open a way to scale-up this method.

## Highlights

• Proof-of-concept method to parallelise phonon microscopy cell elasticity measurements
• A multi-core fibre bundle is used to detect 6 channels in parallel
• A 3-fold increase in acquisition speed is achieved limited by the electronics speed.

## Graphical abstract

**Figure:**
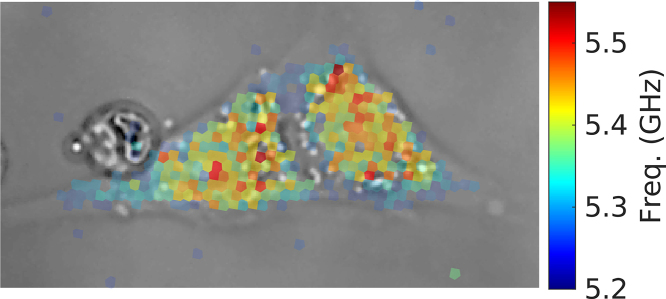

## 1 Introduction

Recently, a suite of microscopy tools is emerging to fulfil new demands in life sciences. These tools complement optical microscopy by providing capabilities such as label-free operation and quantitative contrast. Techniques such as Brillouin microscopy [1], [2] and Raman microscopy [3] are among the most prominent. These techniques offer contrast related to the elasticity of the materials and their molecular content, respectively. Elasticity, in particular, is of growing interest for understanding both healthy and diseased states of cells [4]. Currently, these techniques offer a promise for discovery; however, they exhibit technical drawbacks such as cost, difficulty in implementation and low acquisition speeds.

The ability to have quantitative, label-free contrast often arises from resolving the detected light in the time or frequency domains. Such requirements generally lead to point-by-point scanning and long integration times. This is also a requirement for picosecond laser ultrasound (PLU) techniques such as phonon microscopy, where modulation depths of the signals are on the order of 10^−6^. These techniques use ultrashort pulsed lasers to generate and detect GHz–THz acoustic waves. The emergence of PLU techniques has enabled the study of ultrafast phenomena in biological and non-biological fields. For instance, the electron and phonon dynamics of nanoparticles [5], semiconductors [6] and biological samples [7].

The initial PLU systems resolved the acoustic phenomena by adjusting the timing between the pump and probe pulses using a mechanical delay line [8], [9], [10]. This method allows for extremely fine time resolution that enabled, for instance, the study of thin film made of semiconductor [11], diamond [12], and metal [13]. However, the use of a mechanical delay line comes with a cost of acquisition speed. In this method, each point is averaged before the mechanical stage can move to the next point in the trace, therefore, acquisition speed is typically a few minutes per trace, meaning that imaging remains impractical.

A more recent technique called Asynchronous Optical Sampling (ASOPS) [14] removed the need for a mechanical delay line. Instead, ASOPS replaces it by using two lasers with slightly different repetition rates. The difference in repetition rates, controlled and maintained electronically, provides a rapidly varying delay between the pump and the probe pulse beams that wrap around (typically every 100 μs). With this method, imaging became a reality with applications in biology [15], [16], [17], transducer design [18], among others. However, the imaging speed of ASOPS systems, where single-point acquisition speeds typically range from a few points per second to a few seconds per point depending on the application, remains low compared to optical microscopy. Other reported efforts to increase the acquisition speed of PLU systems involve using linear photodiode arrays to enable multiple simultaneous measurements [19]. However, these operate at low frame rates and require low modulation frequencies for lock-in detection.

In this work, we demonstrate a proof-of-concept to speed up data acquisition in picosecond ultrasonics. Using a fan-out fibre bundle with multiple photodetectors at one of its ends, we can perform multiple measurements simultaneously with only minor modifications to the experimental setup. We demonstrate a gain in acquisition speed, enabled using this technique, measuring the Brillouin frequency shift from phantoms and biological cells. Finally, we discuss factors currently limiting acquisition speed and potential future developments.

## 2 Methods

In this section, we describe the parallel imaging experimental setup, the core position and size calibration, signal processing, image reconstruction, and the preparation of samples, including a binary mask, a polystyrene phantom and a cell sample.

### 2.1 Experimental setup

The phonon microscope was built around a dual Ti:Sapphire (Tsunami Spectra-Physics) laser asynchronous optical sampling system (ASOPS) with 100 fs pulses at 80 MHz repetition rate [20]. This allows the timing of the laser pulses to be precisely controlled and for the time delay between the pulses to be swept from 0–12.5 ns every 200 μs (5 kHz).

**Fig. 1:**
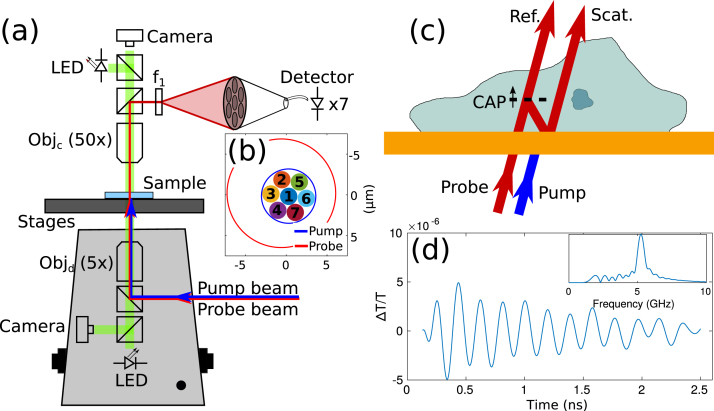
Phonon microscopy setup for parallel imaging. (a) Pump and probe light is delivered through an inverted microscope using a 5x (0.1 NA) objective. The transmitted probe light is collected by a 50x (0.55 NA) objective and re-imaged onto the fibre bundle. (b) Shows the fibre bundle in the sample plane. (c) Light–sound interactions within the cell give rise to Brillouin scattering. The metallic thin-film used for generation and detection allows probe light transmission. (d) Representative time-resolved measurement where the time of flight indicates changes of the relative phase between the reference and scattered beams which occur at different depths, and inset Fourier transform of time trace. The trace was acquired using the centre channel of the multi-core fibre bundle on the cell sample (see Section 3.3).

The pump and probe lasers beams (415 and 780 nm, respectively) were delivered to an inverted microscope (Fig. 1a) and focused on the sample using a 5x objective lens (0.1 NA) which produced about 6.5 and 13 μm spot-sizes for pump and probe, respectively (Fig. 1b). The transmitted laser beams were collected through a 50x objective lens (0.55 NA) and re-imaged by a 400 mm focal length lens onto a multimode core fibre bundle (200 μm core size), with an effective magnification of 100 times (2 μm each core).

In the setup, at the sample (Fig. 1c), the pump laser generates coherent acoustic phonons (CAPs) that propagate through the transparent sample and are optically detected by the probe laser through the effect of time-resolved Brillouin scattering (TRBS) [16]. This is achieved by encoding the amplitude and phase of the phonon with frequency $f_{B}$ onto the time-dependent intensity of the transmitted probe beam. Then, $f_{B}$ can be measured directly by Fourier transforming the time-domain signals (Fig. 1d), allowing measurements of changes in sound velocity along the axial direction (corresponding to instantaneous frequency changes through time).

Traditionally, the TRBS signals are measured pixel-by-pixel using a single photodetector, which makes it slow compared to other microscopy techniques using cameras. To speed-up measurements, the probe light collected after the sample was focused onto a 1-to-7 fan-out fibre bundle (6-cores in a hexagonal grid and 1-core in the centre), and measured by multiple photodetectors simultaneously.

### 2.2 Fibre core calibration

To achieve a lateral resolution similar to single-point TRBS measurements; i.e., 2 μm, the fibre bundle was imaged on the sample plane to match the laser spot-size generated by the 0.1 NA objective lens. Each fibre core was 100 times reduced in size at the sample plane from 200 μm to 2 μm (Fig. 1b), by using the 50x objective lens (0.55 NA) and 400 mm focal length lens.

The position of both pump and probe lasers before scanning were centred with respect to the central core of the fibre bundle by maximising the T (transmittance or DC) and ΔT (AC) signals. Additionally, the position of each core was measured by centroiding the spot at the sample plane when coupling light through the detector side. Both pump and probe laser spot-sizes and the position of the cores are shown in Fig. 1b.

### 2.3 Signal processing and image reconstruction

Time-resolved Brillouin signals, detected by heterodyning of Brillouin scattered and transmitted light, were processed as previously described [16], [21]. Briefly, the signals consist of (1) a coincidence peak caused by electronic dynamics, (2) a slow-decaying thermal response, and (3) Brillouin oscillations. The Brillouin oscillations were extracted from the thermal background by polynomial fit subtraction and the peak frequency in the Fourier domain was found using the FFT algorithm. Both Brillouin oscillations and the frequency domain signal are shown in Fig. 1d, where the position of the peak maximum was used to map the Brillouin frequency.

To reconstruct the images, we considered the spatial distribution of the measurements. With the multi-core fibre bundle set-up, multiple measurements were taken in one traditional point scan position. Here, only 6 channels were measured, whereas the spare one was used for tiling the measurements together. We generated a finer scan mesh of the actual sample image using the core positions and the known scan grid. Then, we assigned regions in the sample space to the nearest core, a process known as Voronoi tessellation. The Voronoi polygons were found using a MATLAB built-in function, and the value assigned to each polygon was simply the measurement value from the seed of each Voronoi cell, a known position from the reconstructed fine mesh.

**Fig. 2:**
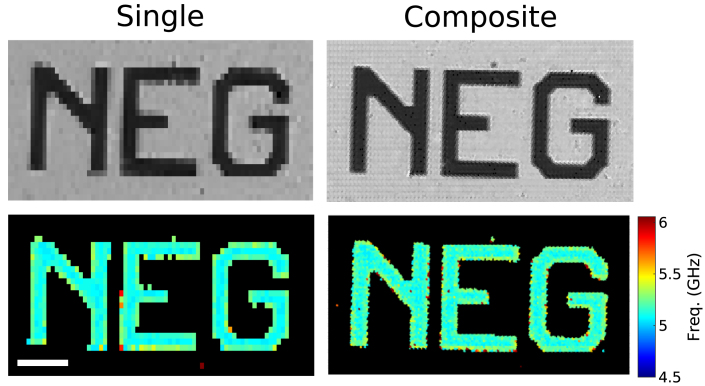
Comparison between imaging with a single channel (left) and composite of 6 parallel channels (right). In both images, the same 4 μm × 6 μm step-size (in x and y-direction, respectively) was used. This shows the increase, up to 6 times, in the number of pixels using the proposed multi-core fibre bundle approach. (Top) optical picture. (Bottom) Brillouin frequency map. The length of the scale bar is 50 μm.

### 2.4 Binary mask sample preparation

The binary mask sample was fabricated using an Electron Beam Lithography (EBL) and thermal evaporation deposition process. An electron beam resists layer (AR-P 6200) and a protecting resist layer (AR-PC 5090) were coated on a cleaned glass coverslip and then after the soft baking process, the electron beam exposure was performed using a Nanobeam nB5 machine with customised GDS designs. The extra protection and conductive layer was required to dissipate charges during electron exposure. Then, deionised water was used to remove the conductive protective layer. Later, 5 nm of indium tin oxide (ITO) and 20 nm of gold (Au) were coated using an n-type thermal evaporator. The gold thickness permits probe light transmission. Finally, the lift-off process was done using AR 600-546.

### 2.5 Polystyrene phantom preparation

Polystyrene phantoms were prepared on a thin-film transducer, made of 20:170:20 nm Au:ITO:Au, respectively, coated onto a sapphire (Al${}_{2}$O${}_{3}$) coverslip (25 mm diameter). Polystyrene beads with 10 μm diameter (PS07001, Bangs Laboratories) were suspended in deionised water at 1000 times dilution. The coverslip was placed on a hot plate at 250 °C and the bead suspension was pipetted on and left for 30 min.

### 2.6 Cell sample preparation

The cell sample was prepared as follows. A human bone fibroblast cell line, MG-63 (ATCC; CRL-1427), was maintained in DMEM (Merck, D6421) supplemented with 2 mM L-glutamine (Merck, G7513), 10% FBS (Merck, F9665), 50 mg/ml penicillin and streptomycin (Merck, P0781). Cells of passage numbers 20–35 were incubated in a humidified atmosphere containing 5% CO2 at 37 °C. Sapphire coverslips (25 mm diameter) deposited with a thin-film transducer (20:170:20 nm Au:ITO:Au) were coated with PLL (Merck, P04707) as described by the manufacturer to aid cell adhesion. Cells were seeded at a density of $10^{5}$ cells per coverslip and grown overnight. After fixation for 10 min with 4% formaldehyde solution (Merck, 1.00496) the coverslips were assembled in a bespoke coverslip holder. This was flushed through with PBS (Merck, D8537) to stop the fixed cells from drying while measurements were taken.

## 3 Results

In this section, we demonstrate the capabilities of the multi-core fibre bundle to acquire Brillouin information using a range of samples: a calibration sample similar to a binary mask (on/off signals), a phantom sample with two very different Brillouin signatures (polystyrene and water), and finally, a cell sample with the demonstration of parallel TRBS signals similar to the current state-of-the-art.

### 3.1 Binary mask sample

This sample consisted of letters made of 20 nm gold thickness and with a line width of 15 μm (see Section 2.4). The sample was immersed in water using a gasket cell chamber and measured with our parallel phonon microscope setup.

Fig. 2 shows a comparison between single channel detection and 6 parallel channels using the fibre bundle setup for detection (Fig. 1). The single channel picture (Fig. 2-left) was acquired with the centre core channel and the parallel detection picture (Fig. 2-right) was acquired using 6 channels from the fibre bundle.

Fig. 2 includes: (top) the optical picture of the scanned area (300 × 160 μm) where the letters (gold) are brighter compared to the background (no gold), and (bottom) the Brillouin frequency map, where 5.3 GHz (Brillouin frequency of water at 780 nm) is detected on the letters.

The sample was scanned using a 4 μm and 6 μm step-size in the x and y direction, respectively, allowing us to tile the image using the spare channel (Ch6, Fig. 1b) and avoid larger empty gaps in the scanning. The 6 channel image was reconstructed following the process explained in Section 2.3.

By using a single fibre channel, the image was formed by 2052 pixels with 4 × 6 μm pixel size, whereas the composite image (using the 6 available channels and the tiling approach), the pixel count was increased by a factor of 6 (12,312 pixels) and smaller pixel size (2 × 2 μm).

### 3.2 Polystyrene phantom sample

After demonstrating the ability to detect Brillouin frequency signals using the fibre bundle setup on selected regions (Fig. 2), we have extended this capability to detect more than one material or multiple Brillouin frequency signatures.

To achieve this, a sample with polystyrene (PS) beads was fabricated (see Section 2.5) and measured in water. In this way, two main characteristic frequency peaks can be detected: 5.2 GHz and 9 GHz, which are the water and polystyrene Brillouin frequencies, respectively.

**Fig. 3:**
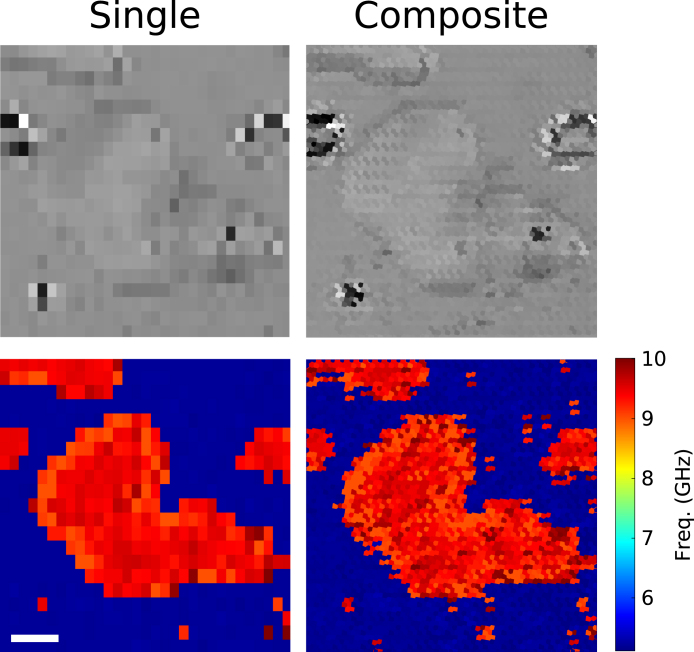
Polystyrene phantom sample surrounded by water. This figure shows a comparison between single core channel (left column) and composite (right column). In both images, the same 4 μm × 6 μm step-size (in x and y-direction, respectively) was used. This shows the increase, up to 6 times, in the number of pixels using the proposed multi-core fibre bundle approach. The top row shows the optical pictures, and the bottom one is the Brillouin frequency map, where water is 5.2 GHz and polystyrene is 9 GHz. The length of the scale bar is 20 μm.

Fig. 3 shows the optical image (top) and the Brillouin frequency map (bottom) of a 120 × 120 μm area of multiple polystyrene beads surrounded by water and scanned with the parallel phonon microscope setup. The different columns show: (left) the data acquired by the centred channel (Ch.1) of the fibre bundle with a step-size of 4 μm in the x-axis and 6 μm in the y-axis, and (right) the composite built by the data acquired by the 6 fibre bundle channels simultaneously.

In terms of pixel size and number of pixels, the single channel image (left-column) is composed by 651 pixels, where each pixel is 4 × 6 μm in size. Therefore, the composite image (right-column) is formed by 3906 pixels (6 × 651 pixels single channel image) with a pixel size of 2 × 2 μm. The discussion of acquisition speed is described in Section 4.

### 3.3 Cell sample

From the previous measured samples, we demonstrated the capabilities of the parallel imaging setup to detect multiple Brillouin signals (e.g. water and polystyrene) and reconstruct images in a similar way as conventional phonon microscopy. In the following, we show the ability to obtain information related to cellular elasticity using our parallel approach with a resolution similar to phonon microscopy (∼2 μm).

**Fig. 4:**
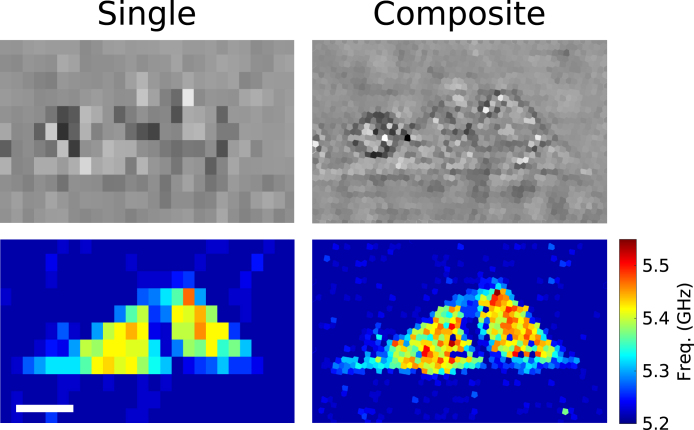
Comparison between single channel (left column) and parallel channel detection (right column). In both images, the same 4 μm × 6 μm step-size (in x and y-direction, respectively) was used. This shows the increase, up to 6 times, in the number of pixels using the proposed multi-core fibre bundle approach. The top row shows the optical pictures, and the bottom one is the Brillouin frequency map. The length of the scale bar is 20 μm.

After fabricating the cell sample (see Section 2.6), we scanned an area of 100 × 60 μm with, again, a step-size of 4 μm in the x-axis and 6 μm in the y-axis direction. This can be seen in the left column of Fig. 4, where top row is the optical picture, and bottom row is the Brillouin frequency map.

From the composite cell image (right column, Fig. 4), it is shown the additional spatial information gained by using the parallel imaging setup. For instance, the stiffer areas or higher frequency values measured here indicate the position of the nucleus.

As before, the number of pixels was increased by a factor of 6 from single channel to composite, from 286 to 1716 pixels. In the following section, the current acquisition speed of our experimental setup is discussed.

## 4 Discussion

The results we presented in the previous sections show the capabilities of the parallel imaging setup to obtain similar resolution and information to conventional phonon microscopy (∼2 μm lateral resolution).

As we described in Section 2.1, the data were acquired through a multi-core fibre bundle and 6 photo-detectors connected to two digital oscilloscopes (using 3 channels per scope). With this arrangement, we should have achieved a 6-fold increase in acquisition speed as 6 points were measured simultaneously; but in reality this is restricted by the hardware limitations. Acquiring the time-traces from the 6 different channels (using the two oscilloscopes) took ∼3.6 s per pixel, whereas, it took ∼1.9 s per pixel when using one channel on a single digital oscilloscope.

It is clear that the main limiting factor is the current speed of the electronics, so a 6-fold increase in acquisition speed, same as the pixel increase factor, could not be reached. However, by using this method, we were able to obtain same images as standard phonon microscopy, while using less scanning steps (by a factor of 6) and getting the same spectral information (shown in Fig. 3, Fig. 4). Taking this into account and knowing the acquisition time difference between using one or two digital oscilloscopes, we were limited to a 3-fold increase in image acquisition speed. With faster acquisition cards, this method can be expanded by using fibre bundles with additional cores.

Excepting the slow-down caused by the oscilloscope, the overall laser exposure for the parallel system compared with the single point system is roughly the same (there are some small additional losses through the fibres, but these can be neglected). The slow-down in averaging means we expose the cells for longer, in this particular system, but at a lower power density (with a lower SNR).

Regarding the SNR, the standard phonon microscope SNR is typically ∼36 when using 1 mW pump and 2 mW probe laser powers (spot-sizes 1 and 2 μm, respectively). On the other hand, the proposed approach using the multi-core fibre bundle, the SNR values are 21, 19, 20, 15, 15 and 16 across the 6 different channels used for detection. Although these values are lower than that for the standard system, the total power density in the proposed approach is also lower, approximately 2.5 times, and some of the light reaching the multi-core fibre bundle is not coupled efficiently (space between cores). However, the composite images we obtained are clearly richer in spatial and spectral information.

Finally, in the multi-core fibre bundle setup, both laser beams are expanded to cover all the seven cores of the fibre bundle (its size is about 6 μm at the sample plane). In a standard phonon microscope setup, pump (415 nm) and probe (780 nm) spot-sizes are usually 1 and 2 μm, whereas here, they are expanded to ∼6.5 μm, and ∼13 μm, respectively. On the other hand, the average power at the sample is increased from 1 to 10 mW for pump laser, and from 2 mW to 30 mW probe laser. Therefore, the total power density at the sample using the multi-core fibre bundle setup is ∼2.5 times lower than that for the standard phonon microscope. However, increasing the average power was not viable as the system could not provide enough pump average power with such a large spot-size, but the current SNR was good. A solution to increase the average power and improve SNR further is to replace the large circular spot (current work) with a line focus for both pump and probe with a multi-core fibre bundle with the cores in a line rather than in a hexagonal grid. In this way, we can increase the number of cores in a line, increase the power density to be similar to the single detector case or average for longer without reducing the acquisition speed, hence, reducing the total laser exposure and improving the biocompatibility greatly.

## 5 Conclusions

In this paper, we have demonstrated a method to acquire multiple measurements simultaneously in picosecond laser ultrasonics techniques but, in particular, phonon microscopy. The proposed setup is simple to implement as it only requires minor changes in the detection path by using a 1-to-7 fan-out fibre bundle with multiple photodetectors. We have obtained similar images as standard phonon microscopy, with same spectral information, while using less scanning steps (by a factor of 6). Thus, we have achieved about 3-fold increase in acquisition speed, which was limited by the electronics speed (i.e., two digital oscilloscopes).

## Declaration of Competing Interest

The authors declare that they have no known competing financial interests or personal relationships that could have appeared to influence the work reported in this paper.

## Data Availability

Data will be made available on request.
